# Bone Age Assessment Empowered with Deep Learning: A Survey, Open Research Challenges and Future Directions

**DOI:** 10.3390/diagnostics10100781

**Published:** 2020-10-03

**Authors:** Muhammad Waqas Nadeem, Hock Guan Goh, Abid Ali, Muzammil Hussain, Muhammad Adnan Khan, Vasaki a/p Ponnusamy

**Affiliations:** 1 Faculty of Information and Communication Technology (FICT), Universiti Tunku Abdul Rahman (UTAR), 31900 Kampar, Perak, Malaysia; vasaki@utar.edu.my; 2 Department of Computer Science, Lahore Garrison University, Lahore 54000, Pakistan; abidali.cs17@gmail.com (A.A.); madnankhan@lgu.edu.pk (M.A.K.); 3 Department of Computer Science, School of Systems and Technology, University of Management and Technology, Lahore 54000, Pakistan; muzammil.hussain@umt.edu.pk

**Keywords:** bone age, deep learning, image processing, health care, survey, segmentation, magnetic resonance images (MRIs)

## Abstract

Deep learning is a quite useful and proliferating technique of machine learning. Various applications, such as medical images analysis, medical images processing, text understanding, and speech recognition, have been using deep learning, and it has been providing rather promising results. Both supervised and unsupervised approaches are being used to extract and learn features as well as for the multi-level representation of pattern recognition and classification. Hence, the way of prediction, recognition, and diagnosis in various domains of healthcare including the abdomen, lung cancer, brain tumor, skeletal bone age assessment, and so on, have been transformed and improved significantly by deep learning. By considering a wide range of deep-learning applications, the main aim of this paper is to present a detailed survey on emerging research of deep-learning models for bone age assessment (e.g., segmentation, prediction, and classification). An enormous number of scientific research publications related to bone age assessment using deep learning are explored, studied, and presented in this survey. Furthermore, the emerging trends of this research domain have been analyzed and discussed. Finally, a critical discussion section on the limitations of deep-learning models has been presented. Open research challenges and future directions in this promising area have been included as well.

## 1. Introduction

The advancement in medical technologies gives more effective and efficient e-health care systems to the medical industry to facilitate the clinical experts for better treatments for patients. E-health care systems are beneficial in various medical domains [1]. Since more computer vision-based biomedical imaging application has gained more importance because these applications provide recognizable information to the radiologists for better treatment [2,3].

Skeletal bone age assessment (BAA) is a mechanism that used for therapeutic investigation [4] and diagnostic of endocrinology problems such as genetic disorders and children’s growth [5], in the field of pediatric radiology [6]. The BAA method is commonly performed by radiological examination of the left hand, due to the discriminant nature of bone ossification stages of the non-dominant hand, and afterward it is compared with chronological age. Magnetic resonance images (MRIs), computed tomography (CT), and X-ray images are most commonly used for the analysis of bone maturity and age. The analysis of bone age from these images modalities is widely used due to simplicity, minimum radiation, and availability of multiple ossification centers. Two clinical methods such as Greulich and Pyle (G&P) and Tanner–Whitehouse (TW) are most commonly employed for BAA [5]. The G&P method used by the 76% radiologists, due to its speed and simplicity, it compares a reference atlas and a whole 15 X-ray scan. This method suffers significantly form inter-observer and intra variabilities [7]. The TW based methods and approaches such as TW2 and TW3 have been used for the analysis of particular bones rather than of whole-body bones as in the G&P method.

In particular, the TW based methods take a set of specific region of interest (ROIs) and then divided it into carpal ROIs and metaphysis/epiphysis ROIs. The ROIs are also divided into discrete stages and each stage is represented with a letter (A, B, C, D,…, I) along with a corresponding numerical score 25 which varies according to sex and race. Afterward, by adding the score of all ROIs the final bone maturity score is obtained. The TW methods used less because they need more time as compared to G&P methods to perform bone age analysis. However, the TW methods give a more effective and accurate performance as compared to G&P methods [8]. The TW methods are also suitable for automation due to their modular structure. Somehow these are automatic medical image analysis methods but need domain expert feedback for a clinical report [9].

Numerous image-processing approaches and techniques have been used for bone age assessment. Segmentation is a basic step in image processing applications that extract the ROIs from different image modalities like MRI, CT, and X-rays [10,11,12,13]. Segmentation of ROIs is an important task for BAA. MRI and X-ray have different features that are used for BAA that includes local histograms [14,15], image textures [16,17], and structure tensor eigenvalues [18,19,20]. 

Deep learning (DL) has gained attention for medical imaging problems. DL-based models and architectures are prominent in bone age segmentation, prediction, and classification. It has performed effectively in the field of medical image analysis, object detection and recognition [21], medical image classification [22,23,24], medical image processing and segmentation [25,26,27]. The deep learning-based models efficiently resolved the problems related to automatic segmentation, classification, and prediction by using MRI and X-ray images. The convolutional neural network (CNN), deep belief network (DBN), and recurrent neural network (RNN) are powerful deep-learning models for image recognition, segmentation, prediction, and classification. The CNN model is most commonly used for segmentation and classification of bone age. Among all the deep learning models, CNN gives effective performance for image segmentation, prediction, and classification. Two-dimensional CNNs (2D-CNNs) and 3D-CNNs [28,29,30,31,32,33] are both used for bone age segmentation. 

The development of deep-learning models and applications toward bone age assessment encouraged us to present an extensive survey on different categories of BAA such as segmentation, classification, and prediction, from applications and methodology-driven perspectives. The survey also describes the summary of large research publications related to BAA in tabular form that help researchers and readers to speedily evaluate the field. The survey also presents a discussion section that covers the successful deep learning development, research challenges, and a summary for future research directions. This survey covers a wide number of recent publications and presents a variety of DL models and applications for BAA. For identification of the most relevant recent contribution “Deep Learning” AND “Bone Age Assessment” query in the title and abstract is performed. In short, the objectives of the presented survey are (a) to present recent deep learning development for bone age assessment, (b) an overview of open research challenges for successful deep-learning models related to BAA, and (c) to highlight the successful and effective DL contribution for BAA.

## 2. Typical Deep-Learning Models

Deep learning is a tremendously hot research area in the community of machine learning that has been presented in science magazines since 2006. Different deep-learning models and techniques have been developed in the last few years. The most typical deep-learning models include CNN, RNN, and DBN that are most commonly used in various fields. Most of the other DL models can be derived from these deep architectures. In this section, these models are briefly reviewed. 

### 2.1. Convolutional Neural Network (CNN)

**Figure 1:**
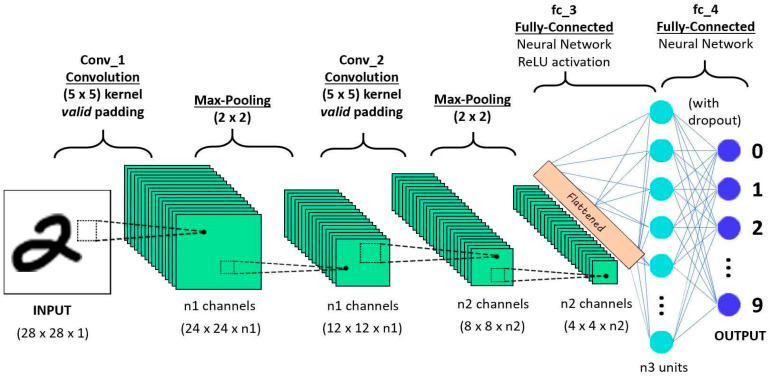
Convolutional neural network [40].

The convolutional neural network is a powerful and commonly useable model that learns the features from a large scale image to perform recognition and classification [34,35,36,37,38,39]. The CNN model has three basic layers such as convolutional, pooling, and fully-connected layers respectively as shown in Figure 1.

The convolutional layer, share the weights by performing convolution operations the weight and the pooling layer reduce the dimensions. For example, suppose x is a two-dimensional image. Firstly, the image x is divided into a sequential input $x=\left\{x_{1},x_{2}\ldots .x_{n}\right\}$. The convolutional layer to share the weights is defined as:

(1) $$y_{j}=f\left(\Sigma_{i}k_{ij}⊗x_{i}+b_{j}\right)$$

The above equation $y_{j}$ denotes the jth output of the convolution layer and $k_{ij}$ indicates the kernel size with the ith input map $x_{i}$ ⊗ indicates the discrete convolutional operator and $b_{j}$ represents the bias. Additionally, f is a non-linear activation function, and it is called a scaled hyperbolic tangent. The pooling layer reduces the dimension of the features map. Average pooling or max pooling operations are typically implemented by the pooling layer. Afterward, a softmax and fully-connected layers are deployed on the top of the layer for recognition and classification. Consequently, the deep convolutional neural network (DCNN) usually consists of many pooling and convolutional layers. In the last few years, the DCNN has achieved an effective performance in various domains such as speech recognition, language processing, and so on [40,41,42,43,44,45].

### 2.2. Deep Belief Network (DBN)

The deep belief network was the first deep-learning model that was trained successfully [46,47]. The DBN is a different form of stacked auto-encoder, and this network is stacked through various restricted Boltzmann machines (RBM). The restricted Boltzmann machine has two layers that include the hidden layer h and visible layer v, as shown in Figure [48,49]. Gibbs samples are used to train parameters by the restricted Boltzmann machine. In particular, conditional probability P (h|v) is utilized in the restricted Boltzmann machine at the hidden layer to compute the value of the respective unit as well as at the visible layer the value of each unit also calculated by conditional probability P (h|v); this mechanism is continuous until the convergence is achieved. RBM has joint distribution for all its units which is defined as:

(2) $$p\left(v,h;\theta \right)=\frac{\exp(-E\left(v,h;\theta \right)}{z}$$

where $Z=\Sigma_{V}\Sigma_{H\;}\exp(-E\left(v,h;\theta \right)$ is utilized for normalization and E is an energy function that used with Bernoulli distribution and computed as:

(3) $$E\left(v,h;\theta \right)=-\overset{I}{\underset{i=1}{\sum }}\;\overset{J}{\underset{j=1}{\sum }}w_{ij}v_{i}h_{j}-\overset{I}{\underset{i=1}{\sum }}b_{i}v_{i}-\overset{J}{\underset{j=1}{\sum }}a_{j}h_{j}$$

In the above equation, i represents the number of visible units and j indicates the number of hidden units.

θ={W,b,a} indicates the set of parameters of the RBM. The computation of sampling probability for the respective unit is described in Equations (4) and (5):

(4) $$P\left(h_{j}=1|v;\theta \right)=f\left(\overset{I}{\underset{i=1}{\sum }}w_{ij}v_{i}+a_{j}\right)$$

(5) $$P\left(h_{j}=1|v;\theta \right)=f\left(\overset{J}{\underset{j=1}{\sum }}w_{ij}v_{i}+b_{j}\right)$$

where f indicates a sigmoid function. The energy function of Gauss–Bernoulli distribution is also used for variation in RBM, which is computed as in [50]:

(6) $$E\left(v,h;\theta \right)=-\overset{I}{\underset{i=1}{\sum }}\;\overset{J}{\underset{j=1}{\sum }}w_{i}v_{i}h_{i}+\frac{1}{2}\overset{I}{\underset{i=1}{\sum }}\left(v_{i}-b_{i}\right)-\overset{J}{\underset{j=1}{\sum }}a_{j}h_{j}$$

Equation (7) computes the conditional probability of every visible unit.

(7) $$p\left(v_{i}=1|h;\theta \right)=N\left(\overset{J}{\underset{j=1}{\sum }}w_{ij}h_{j}+b_{j},1\right)$$

**Figure 2:**
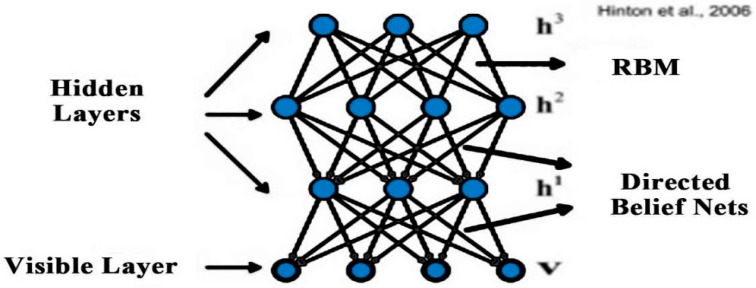
Deep belief network [51].

In the above equation $v_{i}$ denotes the real value, and the mean value of $\sum_{j=1}^{J}w_{ij}h_{j}+b_{j}$ along with variance 1 is used for the satisfaction of Gauss distribution. The transformation of a real variable into a binary variable is performed by RBM with Gauss–Bernoulli distribution. Various RBMs are stacked into a model that is called DBN as shown in Figure 2. The two-stage strategy is used to train the DBN. To train the initial parameters, the pre-training phase is used as an unsupervised manner; a supervised training strategy is used to tune the parameters in the fine-tuning phase to the labeled samples; and a softmax-layer is added on the top of layers. DBN has an extensive range of applications in acoustic modeling [51] and image classification [52,53], and so on [54,55,56].

### 2.3. Recurrent Neural Network (RNN)

**Figure 3:**
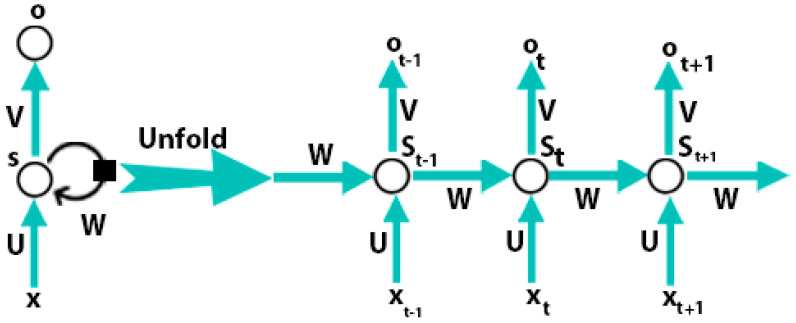
Recurrent neural network [57].

DBN, stacked auto-encoders, and CNN do not deal with problems that are related to time series, so these models are not capable of learning features from time-series data. One natural language sentence is an example of time-series data. Hence, in a sentence every word has a close correlation with other words so, to predict the next word the current word and one or more previous words should be used as input. The feed-forward models do not have the capabilities to store the information of the previous input and these models cannot be performed well for these types of tasks. RNN can learn sequentially form data. The internal state of the neural network store the data of the previous inputs and the RNN model learns the features from that series data. Figure 3 presents the directed cycle which is useful for the construction of a connection between different neurons.

A recurrent neural network consists of input units $\left\{x_{o},x_{1}\ldots \ldots x_{t}\ldots \ldots x_{t+1}\right\}$, output units $\left\{y_{o},y_{1}\ldots \ldots y_{t}\ldots \ldots y_{t+1}\right\}$, and hidden units $\left\{s_{o},s_{1}\ldots \ldots s_{t}\ldots \ldots s_{t+1}\right\}$. Figure 3 is shown that at time step *t*, the RNN takes a current sample $x_{t}$ and the previously hidden representation $s_{t-1}$ as input to achieve the currently hidden representation $s_{t}$:

(8) $$s_{t}=f\left(x_{t},s_{t-1}\right)$$

In the above equation, f is an encoder function. One most commonly useable vanilla of RNN for a time step *t* is described as:

(9) $$s_{t}=f\left(W_{sx},x_{t}+w_{ys}s_{t-1}+b_{s}\right)$$

(10) $$y_{t}=g\left(w_{ys}s_{t-1}+b_{y}\right)$$

where f and g indicate the encoder and decoder, respectively, and $\theta =\left\{W_{sx},W_{ss},b_{s},W_{ys},b_{y}\right\}$ denotes the parameter set. Therefore, the RNN captures the dependencies between the current samples $x_{t}$ with the previous one sample $x_{t-1}$ through the integration of the previously hidden demonstration $s_{t-1}$ into the forward pass. From the theoretical point of view, arbitrary-length dependencies can be capture by RNN. However, the capturing of long-term dependencies by the RNN is a difficult task because of the training of parameters complete by backpropagation with a gradient vanishing strategy. Some other models that prevent a form of gradient exploding or gradient vanishing have been presented to tackle this problem such as long short-term memory (LSTM) [57,58,59,60]. The RNN and its variants have attained great performance in various applications such as machine translation, natural language processing, and speech recognition [61,62,63,64,65].

## 3. Deep-Learning Models for Bone Age Assessment

The bone age assessment process is decided into three stages: segmentation, prediction, and classification. In this section, a comprehensive review of deep-learning models for bone age assessment is presented according to three aspects, i.e., deep-learning models for segmentation, prediction, and classification.

### 3.1. Deep-Learning Models for Bone Segmentation

**Figure 4:**
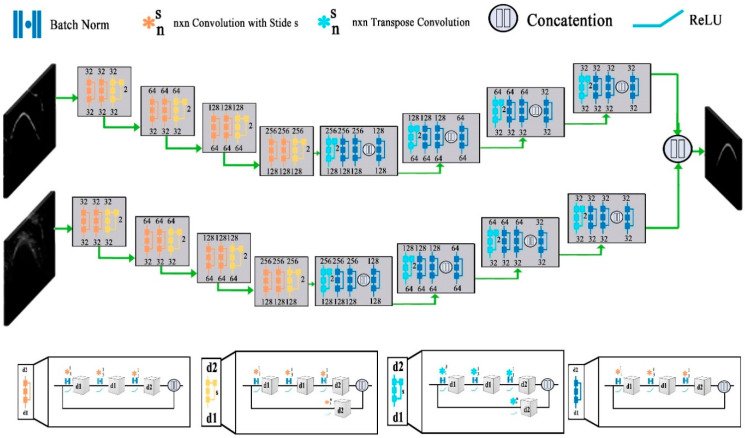
Structure of filter-layer-guided convolutional neural network (CNN) [29].

Bone segmentation is a separation of weak and diffused boundaries of bones that have strong interaction between their adjacent surfaces. Various image modalities such as CT, MRIs, and ultrasound images are used for the segmentation of bones. A 3D surface voxel-based technique is proposed in [66] for the segmentation. This technique consists of three-phase and uses 3D CT images. In Phase-1 the Gaussian standard deviation (GSD) method is applied to locate joined corrector of the bone surface that increases the regular directions of the bone image. In the second phase, the correction of the regular direction is enhanced by updating the values of different parameters of the GSD. In the 3rd phase, the irregular boundaries of the image are modified. This technique is more powerful for tight joint and noisy images. The ultrasound gives real-time, two or three-dimensional images. The interpretation of Ultra Sound (US) based images is hard because of high-level noise, various imaging artifacts, and a very small thickness of the bone surface. Therefore, the segmentation of US-based images is important for bone age assessment. In recent years, a filter-layer-guided CNN is proposed that uses US-based images for the segmentation of bone surface. This method use fusion of feature maps to decrease the variation in the sensitivity of the multi-modal images affected by the artifacts and low image boundary of the bone. Furthermore, the encoders in the CNN-based architecture maps the input image into the low-dimensional latent space and the decoder maps the latent picture into to original space. Firstly, the architecture resizes the US (x, y) input image and its complementary local phase to a standardized 256 × 256 size. In fusion-based CNN the every input image would connect with the independent primary and secondary network. Convolutional blocks process the image in each network and every block consist of many convolutional layers. The architecture divides into four distinct blocks as shown in Figure 4. The d1 and d2 blocks represent the depth of each convolutional layer and blocks represent stride. Furthermore, the skip connection block is used to reduce and restore the channel dimensions. The aforementioned convolutions concatenate with its image to obtain the output of the skip connection block. Finally, in the decoder of each network transpose convolution blocks were implemented to up-sample the feature maps. Similarly, as in the other deep-learning models, the fusion CNN architecture uses batch normalization and rectified linear unit (ReLU) activation at each convolutional layer. Finally, to generate the final segmentation probability distribution sigmoid activation function is used at the output layer [29].

Furthermore, another U-net based encoder-decoder architecture that uses ultrasound images for the segmentation of bone is presented in recent years. This architecture has several contracting convolution layers that are followed by various expanding de-convolutional layers along with a skip connections layer that make the architecture more efficient and effective for the segmentation [67]. Furthermore, the segmentation of the vertebral osteoporosis bones is difficult due to the complexity of the bone shape. A robust FU-net based model was proposed in [68] for the segmentation of vertebral bone. The U-net model is a U-shaped deep learning model that has contraction on the left side and expansion on the right side. This model adds padding in the convolutional layers and uses uniform dimension images for input and output. On the contraction side, the batch normalization and ReLU activation function are applied on each convolutional layer. The architecture takes an overall image of size 128 × 128 of the same dimension and segments the image into two output channels in a probabilistic way.

**Figure 5:**
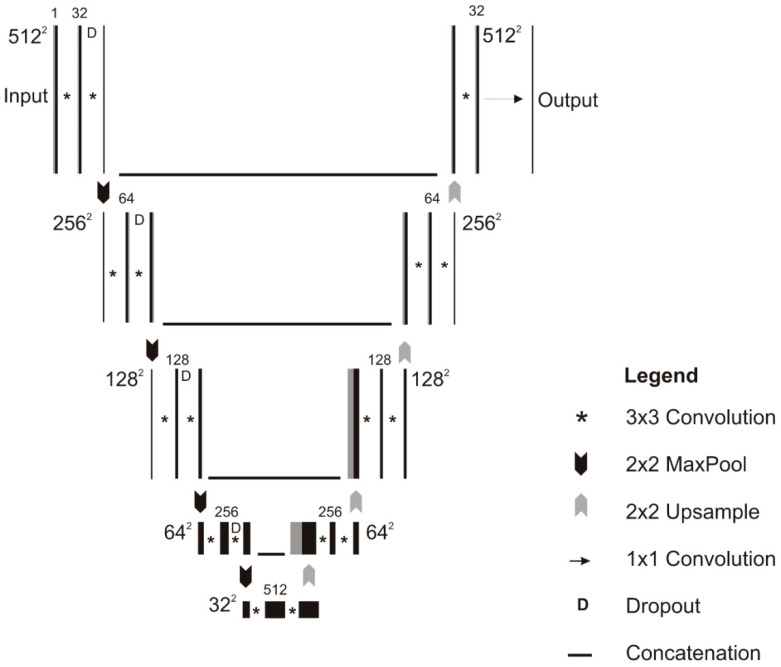
Schematic of the U-net model [69].

Another, U-net CNN as shown in Figure 5 architecture developed by the researchers for the segmentation of bone images. The architecture consists of five convolution layers which accept 512 × 512 input image with four down-sampling layers that convert the image into 32 × 32 × 512 representation along with four up-sampling layers. The dropout 0.20 is applied in down-sizing along with 3 × 3 padded convolutions. At each layer max-pooling with 2 × 2 kernel size and ReLU also applied. The output layer consisted of 1 × 1 convolution that followed by a sigmoid activation function that gives the output score for each class. The U-net CNN is effective and efficient when the size of the data set is limited [69].

In recent years, a fully connected CNN (F-CNN) has been presented for the automatic segmentation of bones instead of manual segmentation. This architecture performs three tasks; firstly, the F-CNN detects the anatomical landmarks of each bone by using the shape model. Then the identified anatomical landmarks are fed to CNN as input for final segmentation. The proposed architecture does not depend on any pre-conceived features or an extensive range of data for training and improvement. It just depends on the size and quality of data that is being fed into the F-CNN. Furthermore, the F-CNN uses CT scans with heterogeneous characteristics form the patients that increase the training performance of the model in recognizing various skeletal patterns [70]. Consequently, another fully convolutional network (FCN) is described in [71] for the automatic localization of bone surface. The FCN model accepts the colored image as input by three different red, green and blue (RGB) channels. The individual channel accepts the original and confident map of the image. The FCN model operates the convolutional filters homogeneously on these three channels. The FCN model is most effective for the segmentation of inter and intra-variation bone surfaces.

Similarly, a multi-feature guided CNN that uses US data was presented in [72]. The model has a pre-enhancing net and modified U-net. The pre-enhancing net phase concatenates the B-mode US input image and three filtered image features to enhance the bone surface, after that U-net performs the segmentation process. Furthermore, the pre-enhancing net enhances the surface of the bone image for better segmentation by performing three tasks that include: (i) local phase tensor image, (ii) local phase bone image, and (iii) bone shadow enhancement. The local phase tensor (LPT(x,y)):LPT(x,y) image is computed by performing even and odd filter response as

(11) $$\begin{array}{c} T_{even}=[H\left(US_{DB}\left(x,y\right)\right][H{\left(US_{DB}\left(x,y\right)\right]}^{T}\\ T_{odd}=-0.5\times \left(\left[\nabla US_{DB}\left(x,y\right)\right][\nabla \nabla^{2}US_{DB}\left(x,y\right){]}^{T}+[\nabla \nabla^{2}US_{DB}\left(x,y\right)\right]\left[\nabla US_{DB}\left(x,y\right){]}^{T}\right) \end{array}$$

In the above equation the $T_{odd}$ and $T_{even}$ represent the asymmetric and symmetric features of US(x,y). $\nabla^{2}$ and H, ∇ represent the Laplacian and gradient operations respectively. Furthermore, to enhance the surface of the bone Log-Gabor filter along with the distance map is also used. $US_{DB}\left(x,y\right)$ represents the resulting image obtained from this operation. The final LPT(x,y) image is obtained using $LPT\left(x,y\right)=\sqrt{T_{even}^{2}+T_{odd}^{2}}\times \cos\left(\emptyset \right)$ where ∅ represents the instantaneous phase obtained from asymmetric and symmetric features of the input image [5]. Furthermore, the local phase of the bone (LP(x,y)):LP(x,y) image is computed using: (LP(x,y))=LPT(x,y)×LPE(x,y)×LwPA(x,y) where LPE(x,y) represents local phase energy features and LwPA(x,y) demonstrates local weighted mean phase angle image features. The monogenic signal theory is used to compute these two features such as $LPE\left(x,y\right)=\sum_{SC}\left|US_{M1}\left(x,y\right)\right|-\sqrt{US_{M2}^{2}\left(x,y\right)+US_{M2}^{3}\left(x,y\right)}$

$$LwPA\left(x,y\right)=arctan\frac{\sum_{SC}US_{M1}\left(x,y\right)}{\sqrt{\sum_{SC}US_{M1}^{2}+\sum_{SC}US_{M2}^{2}\left(x,y\right)}}$$

where $US_{M1},US_{M2},US_{M3}$ denote the three different components of the monogenic signal image $US_{M}\left(x,y\right)$ that measures from LPT(x,y) the image and *sc* represent the number of filters.

The model also enhanced the shadow of a bone (BSE(x,y)):BSE(x,y) image by modeling the interaction of the US signal within tissue as attenuation and scattering.

(12) $$BSE\left(x,y\right)=\left[\left(CM_{LP}\left(x,y\right)-\rho \right)\right]/\left[\max\left(US_{A}\left(x,y\right),\varepsilon \right){]}^{\delta }\right]+\rho$$

where $CM_{LP}\left(x,y\right)$ represents the confident map of the US image and $US_{A}\left(x,y\right)$ maximizes the visibility of the bone features. δ and ρ represent tissue attenuation coefficient and tissue echogenicity respectively. ε is a small constant that is used to escape division by zero.

Finally, the integration of cU-net + PE with the original U-net model gives more effective results as compared to the simple U-net model. The cU-net + PE take more running time because the convolutional layers of the model performed more computation to learn features. Hence the cU-Net + PE for online and off-line applications.

In recent years, an encoder–decoder network (IE2D-net) also presented for segmentation. The IE2D-net imitates the encoding behavior of the convolutional auto-encoder (CAE) in the latent space that uses ground truth as an input to make sure they use of CAE U-net improved decoder component. The enhancement in the U-net architecture and imitation of prior knowledge is better to improve localization capabilities. The IE2D-net consists of three major modules that include U-net subnetwork, CAE, and IE2D-net. The U-net subnetwork extracts the pertinent hierarchical features from the input images. The CAE module enhances the components of the decoder. The IE2D-net combines the imitating encoders that aim to mirror the CAE generated features in latent space, and the CAE decoders improve the hierarchical features for better segmentation. The IE2D-net achieves better accuracy than simple U-net architecture [73]. Table 1 describes the recent deep-learning development for segmentation.

### 3.2. Deep-Learning Models for Prediction of Bone Age

Bone age assessment (BAA) is a fundamental process that is used to evaluate the states of many diseases. The actual BAA process has not significantly changed since the publication of the groundbreaking atlas in 1950 by Greulich and Pyle [76] that was developed between 1931 and 1942 by studying children in Ohio. The BAA process can be implemented by using either the Tanner–Whitehouse (TW2) [77] or Greulich and Pyle (GP) [76] methods. The GP method determines bone age by comparing the patient’s radiograph with an atlas descriptive age. While the TW2 method is based on the scoring mechanism that examines 20 specific bones. In both cases, the BAA process needs considerable time and comprises substantial interrater variabilities, that lead toward clinical challenges when therapy decisions are made based on changes in patients’ BAA. DL [78] gives powerful and efficient models for BAA that overcome these challenges. The DL models replace the conventional models that use manually crafted features for BAA in an automated manner. This section describes the recent devilment in deep learning for BAA.

A fully automated deep-learning model that segments the region of interest, standardizes the image, and processes the input radiographs for the BAA process is presented in [79]. This architecture consists of two parts that include ImageNet pre-trained and a fine-tuned CNN. This model also uses the input occlusion method which creates the attention maps of the input image that reveal which features are used for the training of the model for the BAA process. Finally, the proposed model is deployed in the clinical environment in real-time that gives a much faster interpretation time instead of conventional methods.

Furthermore, skeletal bone age assessment is an extensively famous standard procedure that is used for both growth and disease prediction in the endocrinology area. Furthermore, a CNN and multiple kernel learning (MKL)-based deep automated bone age assessment framework was presented in recent years. The model exploiting the heterogeneous features of the image for bone assessment. Firstly, the visual geometry group (VGG-net) [39] is used to refine the features of the input image. Then the refined features of the image are combined with some other heterogeneous features to make a fused description for the under test object. Support vector regression (SVR) uses the aforementioned heterogeneous features for the estimation of bone age. In the SVR process, the combination of optimal MKL algorithms is used for learning of the model instead of the fixed kernel. This is because the heterogeneous features are from different sources with different similarity notions. The CNN and SVR-based model is effective and gives better performance for the estimation of bone age when the data are in the heterogeneous form [80].

Another, deep-learning based automated bone age estimation model is presented. The model consists of two phases: the features extraction and bone age classification method. The depth neural network (DNN) is utilized to extract local binary pattern (LBP) features and the glutamate cysteine ligase modifier subunit (GCLM) features of the input image. Along with DNN, RCNNs also utilize to locate the key position by creating a sliding window on the input image, the movement of the sliding window along the image gets the potential target areas of the image. The RCNN extract the standard features of the target area and then produces a fixed dimensions output. Finally, the spatial transformer res-net (ST-res net) uses the standard features that produce by RCNN to predict the bone age [81]. In recent years, Greulich–Pyle-based deep learning model developed in [82] for the estimation of bone age, as well as this model, have also validated its feasibility in clinical practice.

Furthermore, a DL and Gaussian process regression (GPR)-based model that observe inter and intra variation in the features of the image for bone age estimation. First, the radiographs are re-scaled to the size of 224 × 224 pixels to train the deep-learning visual geometry group (VGG16) [39] model. During the rescaling process, the aspect ratios of the input image are not altered because they are padded with black pixels. Subsequently, to highlight the surface of the bones in the radiographs, edge enhancement techniques ware also applied in the proposed model. The convolutional layers of the model enhance the pixels of the image where the given matrix E represents the enhancement and e represents the strength of enhancement.

$$E=\left(\begin{array}{ccc} -1 & -1 & -1\\ -1 & e & -1\\ -1 & -1 & -1 \end{array}\right)$$

Afterward, the enhanced image pass-through data augmentation phase which is mandatory to train deep-learning networks because deep learning models require a large amount of data for their training [79]. The model rotates a single radiograph 18 times at [−90, 90] degrees. Hence the sensitivity of the model is enhanced due to rotation and flips of the input image that increases the overall prediction performance of the model instead of using a simple deep-learning model [83]. Another CaffeNet-based Convolution Neural Network model that has low complexity compared to other deep learning models is presented in [84]. The CaffeNet model has numerous edges that are connecting with its neurons, and fixed values of neurons are used. CaffeNet-CNN gives more accuracy when the size of the training data is far smaller.

Furthermore, the LeNet-5 network based on CNN architectures is presented in [85] that accepts a 32 × 32 input image instead of 512 × 512 images to estimate the age of bone. First, the architecture converts the input data into a tf-record format which is faster than the original method. Sliding window operation that may include multiple windows is used by the model to scan the standardized image. Lastly, the maximum connected region algorithm is adopted by the LeNet-5 CNN network to determine the age of the bone. Table 2 describes the recent deep-learning development for prediction.

### 3.3. Deep-Learning Models for Classification

The Greulich and Pyle (GP) and Tanner–Whitehouse (TW2) are traditional clinical methods that are commonly used for BAA and classification. These methods use radiological images for visual examination and classification, and the performance of the examination highly depends on practitioners’ experiences. In recent years, numerous techniques, models, and algorithms are developed that use images-processing techniques to extract explicit features for bone age classification. For example, 15 short bones to encode features of the bone for age estimation. Similarly, in [86] extract features of the bone from seven regions of the interest that includes the carpal bone region and six phalangeal regions for the classification of bone age. However, all these methods and techniques need much effort and are time-consuming. Consequently, the performance of classification extremely depends on hand-crafted features. Therefore, the deep-learning models show prominent performance in bone age classification.

This section briefly describes the recent development in the field of deep learning for bone age classification. DCNNs have had much success in different computer vision problems [87,88]. In [89] a DCNNs model is described for the classification of bone. The DCNNs model extracts the task-relevant, layered and data-driven features automatically from the training data without any feature engineering technique [90]. Due to relatively small samples of training data the DCNN does not give potential solutions. Furthermore, transfer learning is used to train the DCNNs when the size of the data is limited to obtain better performance. First, the DCNNs model use domain knowledge to define different ROIs. Finally, the extracted ROIs and transfer learning are used to perform bone age classification. Similarly, in another study a deep neural network-based model use for the classification of bone. The DNN model to classify the bone on presence skull features on curved maximum intensity projections (CMIP).

A customized CNN that uses a relatively large amount of data as compared to other models is presented in [91]. The model trained from random weight initializations for evaluation and classification of bone age. The customized CNN model consists of a series of convolution matrices that accept vectorized input images and iteratively separate the input image into the target vector space. The main goal of this model is to balance the training parameters for the small size of datasets. The customized CNN model incorporates a combination of residual connections, inception block, and explicit spatial transformation modules. After the initialization of the convolutional layer, a series of residual layers are used in the first portion of the network. The originally residual layers are described in [92,93]; the residual neural networks used by the model to stabilize the gradients during backpropagation, improved the optimization and facilitating greater network depth. Secondly, the inception block introduced by Szegedy et al. [94] also used the customized CNN model to select the optimal filter for input feature maps, and improved the learning rates. The model with residual layers and inception block improve the overall performance of the basic CNN model. Furthermore, a faster-RCNN model with inception-v4 networks is presented in [95,96]. The model is the integration of Tanner–Whitehouse (TW3) and Deep Convolutional Neural Network that used to extract Region of Interest (ROI) for classification. The model explores the expert knowledge from the TW3 method and features engineering from DNN to enhance the accuracy of bone age classification.

Furthermore, most of the studies use transfer learning for bone age classification. A Google Net deep network with a depth of 22 layers was used in [85] for classification. The network was already trained on ImageNet which accepts the final input image and then simply classifies it. The deep convolutional neural with an inception block is used for the training of the classification model. The model reduces the requirement of parameters and increases the number of layers and neurons for effective performance. Table 3 describes the recent deep-learning development for classification.

## 4. Discussion

### 4.1. Overview

A large number of scientific research publications were studied and covered by this survey to elaborate on how DL models achieved effective performance in the field of medical image processing and analysis especially in bone age assessment, segmentation, prediction, and classification. Several deep-learning models are discussed in this research. The CNN is used as a feature extractor in numerous publications. The DCNN- and RNN-based models are also used by [31,39] for BAA. The pre-trained deep learning models are available on different archives and these models download from the repository easily and use for different image modalities. Furthermore, the current models most commonly used hand-crafted image features. In recent years, the researchers preferred end-to-end trained models for medical image processing and analysis. It is also reported that deep-learning models such as CNN, DCNN, and RNN have replaced the traditional hand-crafted ML models and integrated these models into present medical images processing and analysis pipelines. Nemours publications that were covered in this survey follow this mechanism and are being practiced in current standards.

### 4.2. Key Aspects of Successful Deep-Learning Models

After studying a large number of papers, one would expect to be adept to condense the perfect DL model for application areas or individual tasks. The CNN-based models give efficient performance in most bone age assessment applications. We can draw one prominent assumption that is the exact architecture of any model is not a significant determinant for obtaining an effective solution. We have perceived, in various papers i.e., [39,95] that authors have used the same architecture in the model but obtained different results. Furthermore, in [61,85] researchers add more layers in the network of models such as CNN to enhance the performance of the DL model, which is a key aspect that is overlooked in expert knowledge. The authors obtained efficient performance, implement novel preprocessing, and data augmentation techniques along with deep-learning models. In several studies i.e., [83,85], researchers improved the accuracy of the model by adding some normalized pre-processing steps that effectively improved the generalize capabilities of the model without changing the architecture of the model. Consequently, most of the researchers focus on the use of data augmentation strategies and techniques to make the network of models, e.g., the network of the CNN model becomes more robust by using these techniques and improve the performance model. The pre-processing and data augmentation strategies become a key contributor to the effective performance of DL models. Researchers also observed that the designing of a model for a specific task attain betters results as compared to “straightforward” architectures. Multi-scale and multi-view both are cases of the task-specific models. The receptive fields and network input size are fundamental components for designing a network. The increment in patch size should not be changed without the change in receptive fields of the network. If the researchers are domain experts and the model does not give effective performance then there is a need for modification in the network architecture. Furthermore, the performance of the model is also affected by the optimization of different hyper-parameters (e.g., learning rate, dropout rate). There is no best method or technique that currently exists that evaluates the best set of features for empirical exercise. Research also performed and implemented various Bayesian methods that are used for the optimization of hyper-parameters in many medical fields but there is a skill gap in the field of BAA.

### 4.3. Open Research Challenges, Limitations, and Future Directions

The DL models have various unique challenges in the domain of BAA. Firstly, the deep-learning model required large datasets for their training which is a challenging obstacle. In recent years, several X-rays, MRIs, CT, and Premature Atrial Complexes (PACs) systems (i.e., WebPT, MedEZ, Centricity PACs, ImageGrid, and iQ-4CLOUD) have been installed in hospitals that produce a large number of medical images. Digital archives that have image data in well-structured form have been used for a precise goal. It is also perceived that the number of available public datasets for BAA will increase day-to-day. Sophisticated text-mining methods are mandatory while writing reports on structured labels when the automated method is performed. The process of writing reports using automatic structured labeling in the domain of health care, especially in BAA, will become easier in the future. It is also predicted in the future that the use of structured and text-free reports for the training of the models will increase, especially in the field of bone age assessment. The researchers have asked by domain specialists (e.g., radiologists, pathologists) to make task-specific (e.g., segmentation, classification, and prediction) and text–free reports from image data for training DL models. The labeling of bone images requires a high level of expertise and more time and that is why it is a challenging task in the field of BAA. In the training of a 3D network-based model, slice-by-slice annotations are performed by the network which is a challenging and time-consuming task. Effective and efficient training of a model from a limited amount of data is a major limitation of DL models. The additional pre-processing step that includes modeling uncertainty and noise removal is also performed on data before training the model. Some research incorporates label uncertainties directly into loss function, but this is still a challenge. In [30] researchers use 2D segmented data for the training of 3D segmentation models because of the limited amount of training data. Class-imbalance is another problem that is related to training data. For example, several data-augmentation approaches (additive noise, brightness adjustment, image flipping, image cropping, etc.) were used to generate new lesions from the bone image by rotation and scaling the image which may cause class-imbalance. However, most of the DL models in bone age assessment deal with patch-classification, where the network remains unknown as regards the anatomical location of the patch. To solve this problem a solution is for the full image to be stacked into the network of the model and different methods to be used to achieve the learning process of the network, for example, a dice coefficient-based loss function. Since more, if a model has small receptive fields for full image data then this is not beneficent for a model. Due to some limitations such as bandwidth, Graphics Processing Unit (GPU), and limited memory, the feeding of the full image into the network is not feasible because the size of bone images generally in the giga-pixels range. Another challenge for the DL models is that most of the researchers slice the bone input image using the fixed size of the kernel, the results of which may hide or ignore some useful information by the kernel of the model. Very few researchers i.e., [29] used variable kernel size instead of the fix for slicing of the bone image but there is more work needed in this domain for better performance of deep-learning models toward bone age assessment.

## Figures and Tables

**Table 1:** Overview of recent studies for segmentation using deep learning.

Study	Data Set	Method/Model	Proposed Solution	Languages/Libraries/Frameworks/Tools/Software’s for Implementation and Simulation	Evaluation
Faisal Rehman et al. [68]	CSI 2014 and xVertSeg.v1	U-Net	FU-Net based Model	Python using Tensor flow on windows desktop system Intel-(R) i-7 Central Processing Unit (CPU) and 1080 GTX graphics card with GPU memory 8 GB.	Dice Score = 96.4 ± 0.8 ASD (mm) = 0.1 ± 0.05
Ahmed Z. Alsinan et al. [29]	B-mode US images ClariusC3	Convolutional Neural Network	Filter-Layer-Guided CNN	Keras framework and Tensor flow, Intel Xeon CPU at 3.00 GHz, and Nvidia Titan-X GPU with 8 GB of memory, Sonix-Touch	F-Score = 95% Bone Surface Localization Error = 0.2 mm
Robert Hemke et al. [74]	Custom Developed (200 Samples)	Convolutional Neural Network	Deep Convolutional Neural Network-based Model	Python 3.7, Keras library (V2.2.4, https://keras.io), Tensorflow 1.13.1, Multi-GPU (4× NVIDIA Titan Xp units)	Dice score = 0.92 Mean Time to segment one CT image = 0.07 s (GPU), 2.51 s (CPU)
Asaduz Zaman et al. [67]	Custom Developed (1950 Samples)	U-Net	U-Net based Encoder–Decoder Model	Not Mentioned	Dice Score = 0.692 ± 0.011
Sarah Lindgren Belala et al. [70]	Custom Developed at Sahlgrenska University Hospital, Goteborg, Sweden (100 Samples)	CNN	Fully Convolutional Neural Network	Not Mentioned	Sorensen-Dice index (SDI) for Sacrum bone = 0.88%
D. D. Pham et al. [73]	Custom Developed	U-Net	2D Encoder-Decoder based U-Net Model	Tensor flow, GTX 1080 GPU	Dice Score = 73.45 ± 5.93
Haoyan Guo et al. [66]	Custom Developed (212 Samples)	Not Mention	Gaussian Standard Deviation (GSD)	C++, Ubuntu platform, PC with a 2.33 GHz Intel quad-core processor, 8 GB RAM	Dice Overlap Coefficient = 98.06 ± 0.58%
M. Villa et al. [71]	Custom developed US images based (3692 Samples)	Fully Convolutional Networks (FCN)	FCN based Model	Python, Caffe framework	RMSE = 1.32 ± 3.70 mm Mean Recall = 62% Precision = 64% F1 Score = 57% Accuracy = 80% Specificity = 83%
Andre Klein et al. [75]	Custom-developed (6000 Samples)	U-Net	U-Net with Padded Convolutions based Model	MITK, NVIDIA Titan X GPU.	Dice Score = 0.96
Puyang Wang et al. [72]	Custom developed US images based (519 Samples)	CNN	Multi-Feature Guided Convolutional Neural Network (CNN)	MATLAB	Recall = 0.97 Precision = 0.965 F-score = 0.968

**Table 2:** Overview of recent deep-learning development for prediction.

Study	Data Set	Method/Model	Proposed Solution	Languages/Libraries/ Frameworks/ Tools/Software’s Used for Implementation and Simulation	Evaluation
Xu Chen et al. [81]	Custom Developed at Shengjing Hospital of China Medical University (Samples)	Convolutional Neural Network (CNN)	Depth Neural Network, Local Binary Patterns (LBP) features and Glutamate Cysteine Ligase Modifier subunit (GCLM)	Tensor flow	Average Absolute Error = 0.455
Chao Tong et al. [80]	Public Database Digital Hand-Atlas (Samples)	Convolutional Neural Networks (CNNs)	Convolutional Neural Networks (CNNs) and Support Vector Regression (SVR) based Model	Matlab, Keras framework with Tensor Flow	Mean Absolute Error (MAE) = 0.547
Jang Hyung Lee et al. [84]	Radiological Society of North America (RSNA) challenge dataset	CNN	CNN and CaffeNet based Model	Caffe, Tensorflow, Keras, Theano and Torch, Linux Ubuntu OS, NVIDIA GTX 1060 GPU, CUDA library, and CUDNN library.	Concordance Correlation Coefficient = 0.78
Tom Van Steenkiste et al. [83]	Radiological Society of North America challenge dataset (Samples)	Visual Geometry Group (VGG16)echanical Competence and Bone Quality Deve	Visual Geometry Group (VGG16) and Gaussian Process Regression (GPR) based Model	Not Mention	Mean Absolute Difference = 6.80 (−0.94)
Hyunkwang Lee et al. [79]	Custom-developed using open-source software OsiriX and DICOM images (Samples)	CNN	ImageNet pre-trained, fine-tuned convolutional neural network (CNN)	GoogLeNet and Caffe Zoo	Accuracy = 98.56%
Jeong Rye Kim et al. [82]	Custom Developed (Samples)	Deep Neural Network	Greulich-Pyle and Deep Neural Network-Based Model	Not Mention	Root Mean Square Error = 0.42

**Table 3:** Overview of recent deep-learning development for classification.

Study	Data Set	Method/Model	Proposed Solution	Languages/Libraries/ Frameworks/ Tools/Software’s Used for Implementation and Simulation	Evaluation
Jakob Heime et al. [89]	Custom-developed (150 Samples)	Deep Neural Network	Deep Neural Network-based Customized Model	VIDI, COGNEX, Natick, MA, USA	Accuracy = 0.965% Threshold = 0.79% Sensitivity = 91.4% Specificity = 87.5%
Simukayi Mutasa et al. [91]	Digital Hand-Atlas (Samples)	Convolutional Neural Networks (CNNs)	Customized Convolutional Neural Networks with Inception Block	Python Tensor Flow v1.1 library, Ubuntu 16.04 workstation, NVIDIA TITAN X Pascal GPU.	Mean Absolute Error (MAE) = 0.536
Yagang WANG et al. [85]		GoogLeNet		Matlab	Accuracy = 94.4%
Toan Duc Bui et al. [95]	Public Dataset Digital Hand-Atlas (DHA) (1375 Samples)	Deep Convolutional Networks (DNNs)	Deep Convolutional Networks (DNNs) and Tanner Whitehouse (TW3) based Model	Not Mentioned	MAE = 0.59 RMS = 0.76
Jianlong Zhou et al. [97]	Digital Hand-Atlas	Convolutional Neural Networks	Convolutional Neural Networks and Transfer Learning Based Model	Not Mentioned	MAE = 0.72
